# Successful treatment of a B/T MPAL patient by chemo-free treatment with venetoclax, azacitidine, and blinatumomab

**DOI:** 10.1007/s00277-024-05644-9

**Published:** 2024-02-17

**Authors:** Shaoyu Liu, Qingya Cui, Mengyun Li, Zheng Li, Sifan Chen, Depei Wu, Xiaowen Tang

**Affiliations:** 1 National Clinical Research Center for Hematologic Diseases, Jiangsu Institute of Hematology, The First Affiliated Hospital of Soochow University, Suzhou, China; 2 Collaborative Innovation Center of Hematology, Institute of Blood and Marrow Transplantation, Soochow University, Suzhou, China

**Keywords:** Chemo-free treatment, B/T MPAL, Venetoclax, Blinatumomab

## Abstract

B/T mixed phenotype acute leukemia (MPAL), which represents only 2–3% of all MPAL cases, is classified as a high-risk leukemia subtype. Adults diagnosed with B/T MPAL have a notably low 3-year survival rate, estimated at 20–40%. The rarity and undercharacterization of B/T MPAL present substantial challenges in identifying an optimal treatment protocol. This report aims to shed light on this issue by presenting a case in which a patient with a complex karyotype was treated using a combination of venetoclax, azacitidine, and blinatumomab. This novel, chemo-free regimen resulted in the patient achieving both hematologic and molecular complete remission, with no severe organ or hematological toxicity observed. Notably, the patient continued to maintain molecular remission for 1 year following the transplantation. Based on these findings, the combination of venetoclax, azacitidine, and blinatumomab could be considered a potential therapeutic approach for B/T MPAL patients, meriting further investigation.

**Supplementary Information:**

The online version contains supplementary material available at 10.1007/s00277-024-05644-9.

## Background

B/T mixed phenotype acute leukemia (MPAL) is a rare and prognostically unfavorable leukemia subtype characterized by simultaneous immunophenotypic features of both B and T lymphoid lineages. It demonstrates significantly inferior clinical outcomes, with median overall survival (OS) and disease-free survival (DFS) recorded at 8 and 6 months, respectively [1–3]. Furthermore, the 3-year survival rate for adults with MPAL is alarmingly low, ranging from 20 to 40% [4, 5].

The therapeutic landscape for MPAL predominantly relies on standard combination chemotherapy, which includes acute lymphoblastic leukemia (ALL) regimen, acute myeloid leukemia (AML) regimen, or “hybrid” regimens that integrate elements from both. However, the establishment of an optimal treatment strategy remains a significant clinical challenge. Research indicates that while ALL-like induction chemotherapy results in higher rates of complete remission (CR) for MPAL, it fails to confer a survival advantage in terms of long-term OS. Additionally, the propensity for high relapse rates and the substantially reduced CR rates post-relapse in MPAL may be linked to the presence of minimal residual disease (MRD) and subclonal populations [6–10].

In the absence of an international treatment consensus, targeted therapies like the BCL-2 inhibitor venetoclax and blinatumomab, a bispecific T-cell engaging antibody construct targeting CD19, have emerged as promising therapeutic options. Empirical evidence supports their sensitivity and potential efficacy in MPAL treatment [11, 12].

Herein, we present the first report, as we know, of using a chemo-free regimen in a B/T MPAL patient, comprising a combination of venetoclax, azacitidine, and blinatumomab. This innovative approach aims to address the unmet clinical need in MPAL treatment, opening avenues for potential new treatment strategies.

## Case presentation

**Fig. 1:**
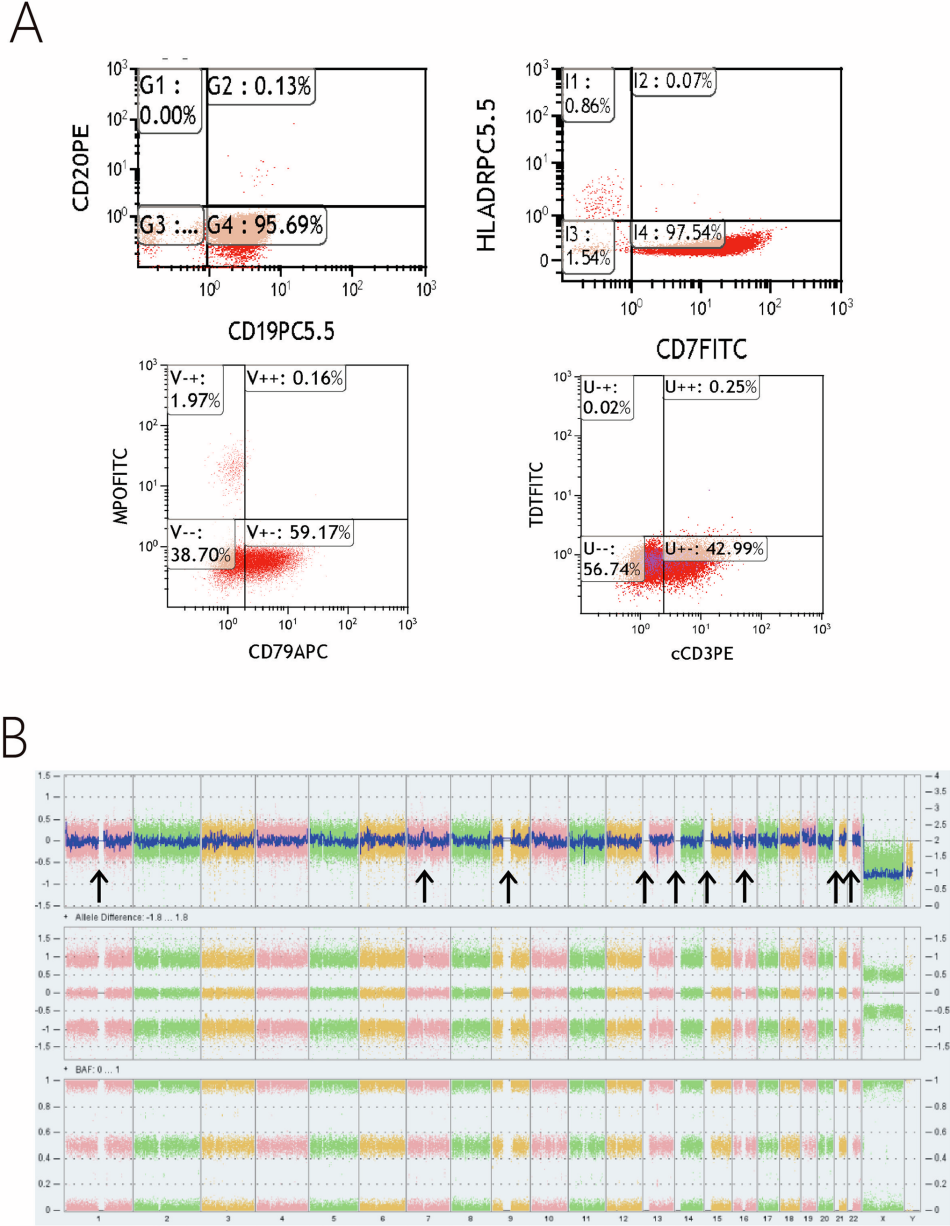
**A** Flow cytometry analysis of the immunophenotype indicated a probable diagnosis of mixed B/T leukemia. **B** Cytoscan analysis revealed complex karyotypes in the patient, suggesting the potential presence of subclones

A 47-year-old male presented with chest tightness and shortness of breath with palpitations after activity for 3 months. The patient had a previous history of hypertension and diabetes mellitus. Complete blood count: WBC 4.82 × 10^9^/L,NE 1.76 × 10^9^/L, HGB 80 g/L, PLT 56 × 10^9^/L. Bone marrow aspiration showed 24% of morphological blast population. Flow cytometry showed 42.5% blasts with a mixed expression of B/T lymphoid lineage, including CD19: 95.69%, CD79a: 59.17%, cCD3 + : 42.99% (Fig. 1A). Karyotype: 46, XY. Next Generation Sequencing (NGS): ASXL1/RUNX1/EZH2/NOTCH1/JAK1/JAK3/NRAS mutations. RNA-sequence: negative. Fluorescence in situ hybridization(FISH):BCR/ABL and ABL1/ABL2/ JAK2/CRLF2/CSF1R/EPOR/PDGFR were all negative. Cytoscan: multiple CNVs were detected including 1p36 gain (2.8 Mb), 1p36 loss (5.9 Mb), 1p36 loss (2.4 Mb), 2q31 loss (3.7 Mb), 2q31q32 loss (7.8 Mb), 2p23p22 loss (2.9 Mb), 5q35 gain (1.7 Mb), 5q32q33 loss (7.9 Mb), 6p22 gain (4 Mb), 10p15p14 gain (9.1 Mb), 13q14 loss (1.6 Mb), 13q14 loss (2.1 Mb), 17q25 gain (2.5 Mb), 19q13 gain (2.1 Mb), 19p12p11 gain (3.9 Mb), and 22q13 gain (1.3 Mb). Sixteen CNVs were present in nine chromosomes, all with fragment sizes > 1 Mb, seven of them > 3 Mb, which suggested complex karyotypes (Fig. 1B). Additionally, the difference between the proportion of 13q-abnormal clones and other abnormalities in this patient was over 30%, suggesting the possible existence of subclones.

**Fig. 2:**
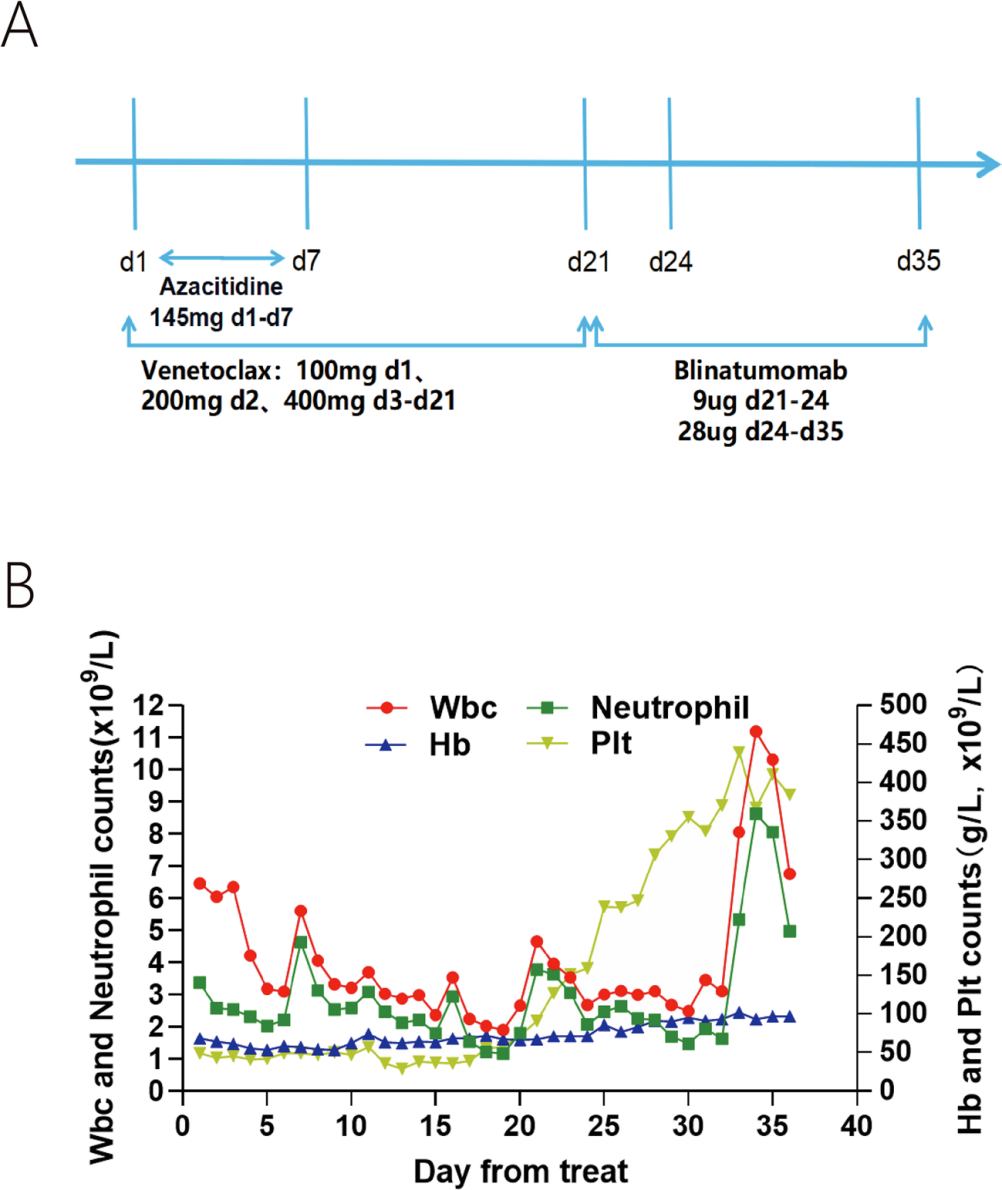
**A** Treatment protocol: The patient was administered a regimen comprising venetoclax (100 mg on day 1, 200 mg on day 2, and 400 mg from day 3 to day 21), azacitidine (145 mg daily from day 1 to day 7), and BLINCYTO (9 µg from day 21 to day 24, escalated to 28 µg from day 24 to day 35). **B** Hematological profile: A graphical representation delineates the temporal changes in the patient’s blood counts throughout the treatment course

**Fig. 3:**
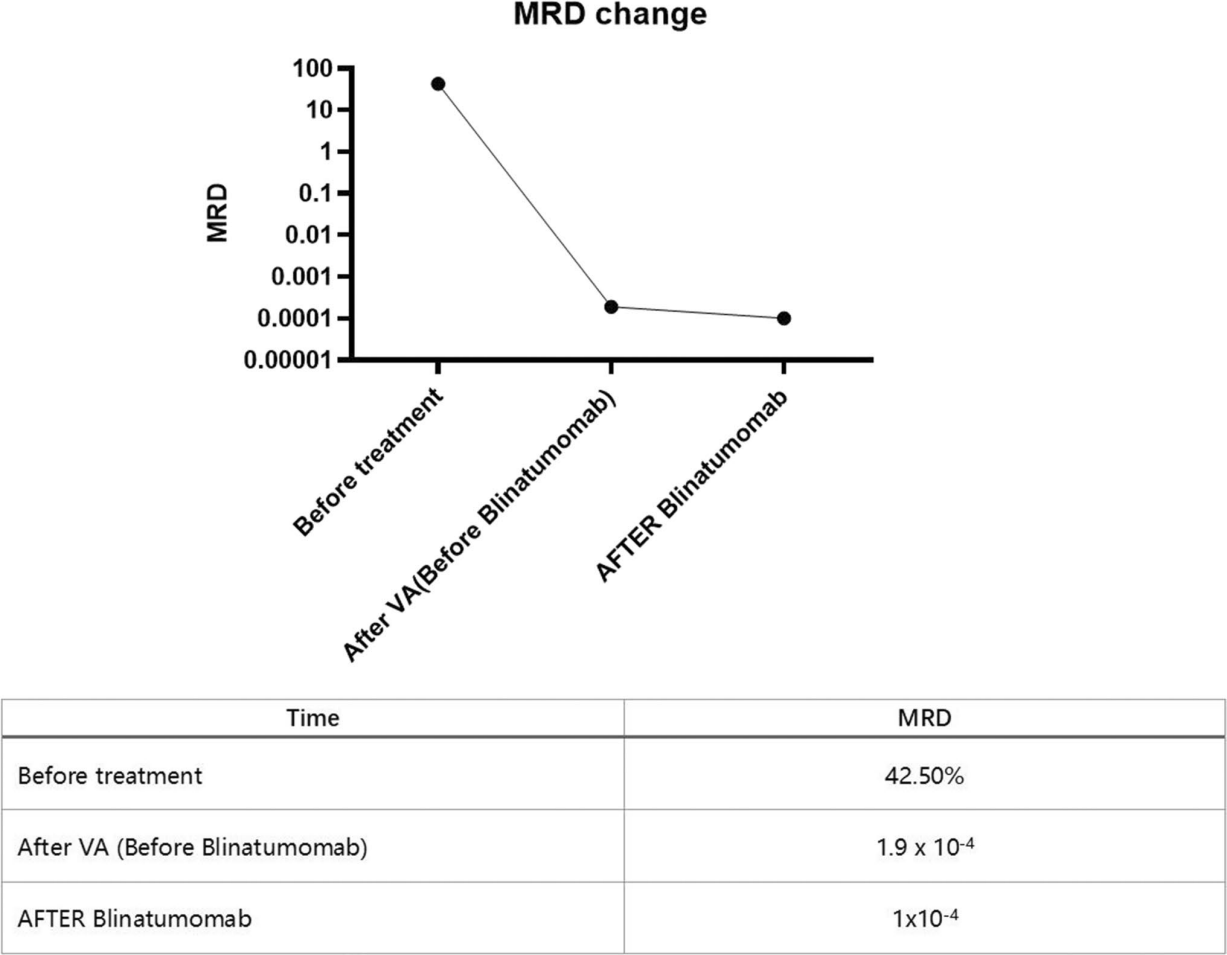
The MRD changes during the chemo-free treatment with venetoclax, azacitidine, and blinatumomab

The patient was treated with venetoclax and azacitidine to reduce tumor load as follows: venetoclax: 100 mg d1, 200 mg d2, 400 mg d3–d21; azacitidine: 75 mg/m2/d IH d1–d7 (Fig. 2A). Then he achieved hematologic CR and the MRD was 1.9 × 10^−4^ (Fig. 3). Considering the presence of subclones and complex karyotypes, blinatumomab 9 ug d1–3, 28 ug d4–d14 was given after the end of the VA (venetoclax and azacitidine) regimen. There were no significant adverse therapeutic effects during the whole treatment with the neutropenia status for 3 days and the platelets consistently above 30 × 10^9^/L (Fig. 2B). Bone marrow aspiration after the end of treatment indicated that the patient achieved hematologic and molecular CR which means the mutations found initially and the cytoscan turned negative. The MRD was < 1.0 × 10^−4^, which suggested the blinatumomab could reduce undetectable MRD (Fig. 3). Besides, no primitive leukemia cells were found in the cerebrospinal fluid (CSF) from the time of diagnosis until now. Peripheral stem cells from an unrelated donor were subsequently transplanted to patient after two consolidation therapy and the modified Bu-Cy conditioning regimen (Supplementary Material). Cyclosporine A (CsA), short-course methotrexate (MTX), mycophenolate mofetil (MMF), and rabbit antithymocyte globulin (Thymoglobulin™) were adopted for graft-versus-host disease (GVHD) prophylaxis (Supplementary Material 2 for usage). Now the patient has remained without evidence of disease for 1 year after transplantation.

## Discussion and conclusions

The B-cell lymphoma 2 (BCL-2) protein family plays a pivotal role as regulators of the intrinsic mitochondrial apoptotic pathway through direct protein–protein interactions. Enhanced expression of BCL-2 family proteins has been observed in leukemia blasts, with a significant proportion of leukemia stem cells exhibiting abnormally high levels of BCL-2, relying on it for survival. Moreover, heightened expression of BCL-2 proteins correlates with disease progression and chemotherapeutic resistance in leukemia patients [13, 14]. Venetoclax, a potent and selective oral BCL-2 inhibitor, has emerged as a new standard in the treatment of AML in elderly and unfit patients when combined with hypomethylating agents like azacitidine, markedly improving overall survival and quality of life [15, 16]. Studies also indicate the potential benefits of the synergistic effects of venetoclax with demethylating agents for ALL [17, 18]. Furthermore, blinatumomab, a bispecific monoclonal antibody, has shown efficacy in enhancing response, survival, and rates of undetectable MRD in B-ALL [19, 20].

B/T MPAL, a rare leukemia subtype meeting criteria for both B-cell and T-cell lineages, presents a grim prognosis with a 3-year survival rate of merely 20–40%. While studies suggest that ALL-like induction chemotherapy yields higher CR rates compared to AML-like regimens for MPAL, it does not confer a long-term OS advantage. However, allogeneic hematopoietic stem cell transplantation (Allo-HSCT) post-chemotherapy significantly enhances survival, especially for patients achieving CR before transplantation [21, 22].

Motivated by these challenges, we explored a chemo-free regimen for B/T MPAL, aiming to achieve CR1 and minimize adverse effects for subsequent HSCT. Herein, we report, to our knowledge, the first successful application of a chemo-free regimen in B/T MPAL, comprising venetoclax, azacitidine, and blinatumomab. Given the lack of consensus on MPAL treatment, transitioning from retrospective studies to prospective trials is imperative. Our report on the effective treatment of this B/T MPAL case may provide valuable insights for the management of similar patients and could potentially influence future therapeutic strategies for this rare leukemia subtype.

### Supplementary Information

Below is the link to the electronic supplementary material.

Supplementary file1 (XLS 33 KB)

Supplementary file2 (DOC 14 KB)

## Data Availability

No datasets were generated or analysed during the current study.

## Abbreviations

*ALL*: Acute lymphoblastic leukemia
*Allo-HSCT*: Allogeneic hematopoietic stem cell transplantation
*AML*: Acute myeloid leukemia
*BCL-2*: B-cell lymphoma 2
*CR*: Complete remission
*FISH*: Fluorescence in situ hybridization (FISH)
*MPAL*: Mixed phenotype acute leukemia
*MRD*: Minimal residual disease
*NGS*: Next generation sequencing (NGS)
*OS*: Overall survival
*VA*: Venetoclax and azacitidine
*CSF*: Cerebrospinal fluid
*CsA*: Cyclosporine
*MTX*: Methotrexate
*MMF*: Mycophenolate mofetil
*GVHD*: Graft-versus-host disease

## References

[1] Matutes E, Pickl WF, Van'T Veer M (2011). Mixed-phenotype acute leukemia: clinical and laboratory features and outcome in 100 patients defined according to the WHO 2008 classification. Blood.

[2] Weinberg OK, Arber DA (2010). Mixed-phenotype acute leukemia: historical overview and a new definition. Leukemia.

[3] Gerr H, Zimmermann M, Schrappe M, Reinhardt D (2010). Acute leukemias of ambiguous lineage in children: characterization, prognosis and therapy recommendations. Br J Haematol.

[4] Alexander TB, Gu Z, Mullighan CG (2018). The genetic basis and cell of origin of mixed phenotype acute leukemia. Nature.

[5] Rubnitz JE, Onciu M, Pui CH (2009). Acute mixed lineage leukemia in children: the experience of St Jude Children’s Research Hospital. Blood.

[6] Maruffi M, Sposto R, Oberley MJ, Kysh L (2018). Therapy for children and adults with mixed phenotype acute leukemia: a systematic review and meta-analysis. Leukemia.

[7] Rasekh EO, Osman R, Ibraheem D (2021). Acute lymphoblastic leukemia–like treatment regimen provides better response in mixed phenotype acute leukemia: a comparative study between adults and pediatric MPAL patients. Ann Hematol.

[8] Beldjord K, Chevret S, Asnafi V (2014). Oncogenetics and minimal residual disease are independent outcome predictors in adult patients with acute lymphoblastic leukemia. Blood.

[9] Orgel E, Oberley M, Li S (2016). Predictive value of minimal residual disease in WHO2016-defined mixed phenotype acute leukemia (MPAL). Blood.

[10] Yu J, Li Y, Xing H (2019). Clinical characteristics and outcome of biphenotypic acute leukemia: 10 cases report and literature review. Blood.

[11] Liu K, Li Y, Qiu S (2021). Efficacy of combination of venetoclax with azacitidine or chemotherapy in refractory/relapse acute leukemias of ambiguous lineage, not otherwise specified. Exp Hematol Oncol.

[12] Brethon B, Lainey E, Caye-Eude A (2021). Case report: targeting 2 antigens as a promising strategy in mixed phenotype acute leukemia: combination of blinatumomab with gemtuzumab ozogamicin in an infant with a KMT2A-rearranged leukemia. Front Oncol.

[13] Courtney D, DiNardo MD, Brian A, Jonas MD (2020). Azacitidine and venetoclax in previouslyuntreated acute myeloid leukemia. N Engl J Med.

[14] Seymour JF, Kipps TJ (2018). Venetoclax–rituximab in relapsed or refractory chronic lymphocytic leukemia. N Engl J Med.

[15] DiNardo CD, Pratz K, Pullarkat V (2019). Venetoclax combined with decitabine or azacitidine in treatment-naive, elderly patients with acute myeloid leukemia. Blood.

[16] DiNardo CD, Jonas BA, Pullarkat V (2020). Azacitidine and venetoclax in previously untreated acute myeloid leukemia. N Engl J Med.

[17] Aumann S, Shaulov A, Haran A (2022). The emerging role of venetoclax-based treatments in acute lymphoblastic leukemia. Int J Mol Sci.

[18] Gibson A, Trabal A, McCall D (2021). Venetoclax for children and adolescents with acute lymphoblastic leukemia and lymphoblastic lymphoma. Cancers (Basel).

[19] Kantarjian H, Stein A, Gökbuget N (2017). Blinatumomab versus chemotherapy for advanced acute lymphoblastic leukemia. N Engl J Med.

[20] Gökbuget N, Dombret H (2018). Blinatumomab for minimal residual disease in adults with B-cell precursor acute lymphoblastic leukemia. Blood.

[21] Munker R, Brazauskas R (2016). Allogeneic hematopoietic cell transplantation for patients with mixed phenotype acute leukemia. Biol Blood Marrow Transplant.

[22] Tian H, Xu Y (2016). Comparison of outcomes in mixed phenotype acute leukemia patients treated with chemotherapy and stem cell transplantation versus chemotherapy alone. Leukemia Research.

