# Supplementary material for: Successful treatment of a B/T MPAL patient by chemo-free treatment with venetoclax, azacitidine, and blinatumomab
Source: Ann Hematol. 2024 Feb 17;103(4):1397–402. doi: 10.1007/s00277-024-05644-9 (PMC10940381; doi:10.1007/s00277-024-05644-9)
Supplement: Supplementary file 2 — Supplementary file2 (DOC 14 KB) [file 277_2024_5644_MOESM2_ESM.doc]

The specific usage of conditioning regimen and GvHD prophylaxys adopted for transplantation

The modified Bu-Cy regimen (Me-CCNU 250 mg/m2 /d orally on day −9, cytarabine 2 g/m^2^ i.v. on day −8, busulfan 0.8 mg/kg i.v. every 6 h on days −7 to −5 (12 doses), and cyclophosphamide 1.8 g/ m^2^ /d i.v. on days −4 and −3) was adopted for transplantation.About GVHD prophylaxys,from day −9, 0.5 g of MMF was administered orally every 12 h, and was discontinued on day+28 post-transplant. Short-course MTX was administered on day+1 (15 mg/m^2^ ),+3, +6, and +11 (10 mg/m^2^ ). Rabbit-ATG (2.5 mg/kg/d, i.v.) was used from day −5 to −2.
